# Strain specific motility patterns and surface adhesion of virulent and probiotic *Escherichia coli*

**DOI:** 10.1038/s41598-021-04592-y

**Published:** 2022-01-12

**Authors:** M. M. Abdulkadieva, E. V. Sysolyatina, E. V. Vasilieva, A. I. Gusarov, P. A. Domnin, D. A. Slonova, Y. M. Stanishevskiy, M. M. Vasiliev, O. F. Petrov, S. A. Ermolaeva

**Affiliations:** 1 Department of Dusty Plasma, Joint Institute for High Temperatures, Russian Academy of Sciences, Moscow, Russia; 2 Peoples’ Friendship, University of Russia (RUDN University), Moscow, Russia; 3 Laboratory of Ecology of Pathogenic Bacteria, N. F. Gamaleya National Research Centre of Epidemiology and Microbiology, Ministry of Health of the Russian Federation, Moscow, Russia; 4 National Research University “Moscow Power Engineering Institute”, Moscow, Russia; 5 Laboratory of Molecular Microbiology, Center of Life Sciences, Skolkovo Institute of Science and Technology, Moscow, Russia; 6 Laboratory of Molecular Immunology, Dmitry Rogachev National Medical Research Center of Pediatric Hematology, Oncology and Immunology, Russian Ministry of Health, Moscow, Russia

**Keywords:** Biophysics, Motility, Microbiology, Clinical microbiology

## Abstract

Bacterial motility provides the ability for bacterial dissemination and surface exploration, apart from a choice between surface colonisation and further motion. In this study, we characterised the movement trajectories of pathogenic and probiotic *Escherichia coli* strains (ATCC43890 and M17, respectively) at the landing stage (i.e., leaving the bulk and approaching the surface) and its correlation with adhesion patterns and efficiency. A poorly motile strain JM109 was used as a control. Using specially designed and manufactured microfluidic chambers, we found that the motion behaviour near surfaces drastically varied between the strains, correlating with adhesion patterns. We consider two bacterial strategies for effective surface colonisation: horizontal and vertical, based on the obtained results. The horizontal strategy demonstrated by the M17 strain is characterised by collective directed movements within the horizontal layer during a relatively long period and non-uniform adhesion patterns, suggesting co-dependence of bacteria in the course of adhesion. The vertical strategy demonstrated by the pathogenic ATCC43890 strain implies the individual movement of bacteria mainly in the vertical direction, a faster transition from bulk to near-surface swimming, and independent bacterial behaviour during adhesion, providing a uniform distribution over the surface.

## Introduction

Flagella-dependent motility characteristics of many bacterial species are important features providing effective dissemination and occupation of novel environments. Flagella themselves provide motility and are also involved in interactions with surfaces, performing the initial steps in surface adherence and biofilm formation. Some pathogenic species use flagella for host tissue colonization^3–6^.

According to the model proposed by Misselwitz et al., interactions of motile bacteria with the cell surface are carried out in four stages: (1) landing (exit of the bacterium from the bulk), (2) near-surface swimming (NSS), (3) stopping (interactions between bacterial receptors and the surface), and (4) taking off (if the interactions are weak) or adhesion, which happens rarely^7^. Experimental observations on widely used model microorganisms, such as the Gram-negative bacterium *Escherichia coli*, established that trajectories of bacterial movement also depend on the distance from the solid surface. At a distance of 20–120 µm (‘bulk’), about 70% bacteria show ‘run and tumble’ trajectories that are a number of broken lines, and movement of the rest 30% is characterized by smoother trajectories with smaller angles of rotation, named ‘slow random walk’^8^. Bacteria that locate closer to the glass at a distance of equal or less than 20 μm, i.e., at the «landing» stage, rotate (‘tumble’) less often, and their reorientation angles are about 34% smaller than the bacteria located in the bulk of the suspension. The direction of bacterial movement at a distance of 10–20 μm is often parallel to the surface^8^. Motile species at close vicinity to the surface (a distance of less than 5 μm) demonstrate near-surface swimming that includes such a typical trajectory as long circular movement.

Many authors have suggested theoretical models of the described trajectories to provide arguments for different movement types^9–11^. To explain the biological sense of the ‘unproductive’ NSS circular motion demonstrated by the enterohemorrhagic *E. coli* O157:H7 strain, Ipina et al. proposed a mathematical model based on short-term stops and attachment of bacteria at various points of the trajectory, important for effective surface colonisation and, consequently, survival in the environment and inside the host^12^. Other researchers have demonstrated that bacteria can interact with surfaces through ‘head’ or ‘tail’ collisions, the latter being more effective for adhesion^13^.

Bacterial movement has been studied in different bacterial strains and species, and although general movement patterns describe a majority of observations, a particular movement style may prevail in one bacterial species but can be rare in another. Meanwhile, bacteria are highly diverse, and the differences between strains within a single species might substantially affect motility. This interspecies difference is important for understanding the mechanisms underlying the strain-specific potential to survive within specific ecological niches. In particular, strains of well-known species such as *E*. *coli* are morphologically and physiologically very different. For example, the laboratory strain of *E. coli* JM109 is flagellated and forms biofilms well, but it is poorly motile due to a deletion of the *rec*A gene, necessary for standard flagellar rotation switching^14–17^. Virulent toxin-producing *E. coli* strains belonging to the serotype O157:H7 are flagellated, motile, and successfully colonise the host intestine but are poor biofilm producers under experimental conditions^18–21^ Comparison of different strains under the same conditions is required to understand how motility patterns affect bacterial strain-specific potential to colonise different surfaces. In this study, we compared movement trajectories of pathogenic and probiotic *E. coli* strains (ATCC43890 and M17, respectively) at the landing stage (i.e., leaving the bulk and approaching the surface) and its correlation with adhesion patterns and efficiency.

## Experimental results

### Probiotic and virulent E. coli strains demonstrated similar macroscopic motility

Three *E. coli* strains were used, including the motile commensal probiotic strain M17, the motile pathogenic O157:H7 strain ATCC43890, and almost non-motile, but flagellated laboratory strain JM109 as a negative control^22^.

**Figure 1:**
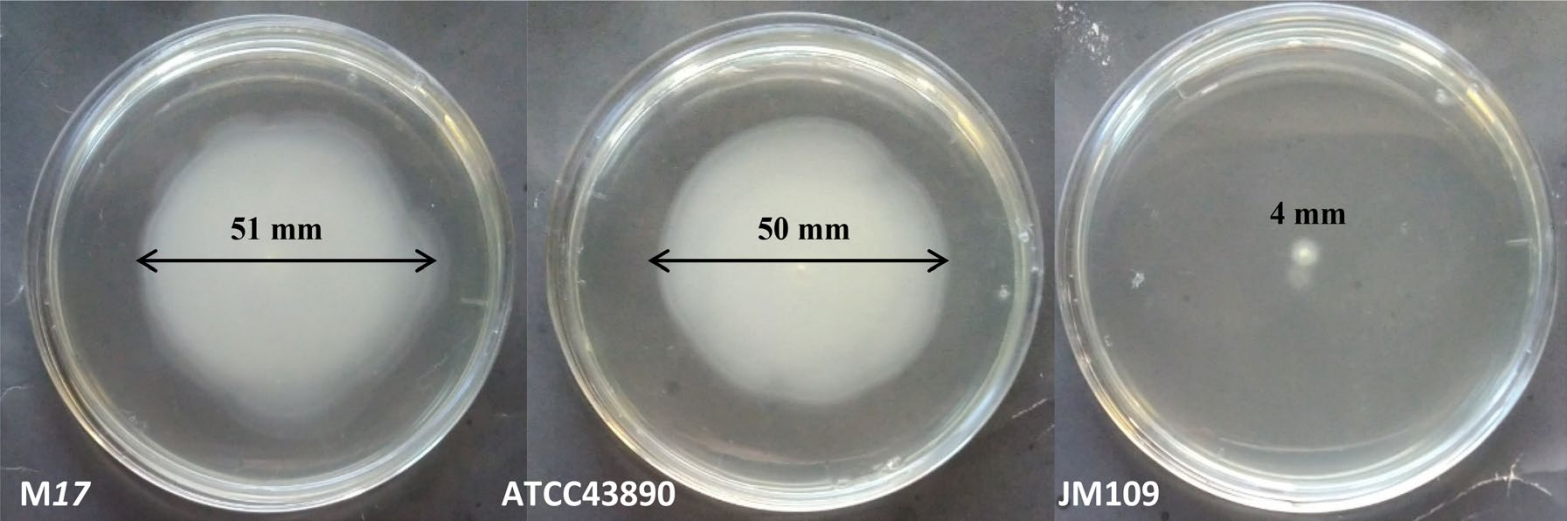
Motility of *E. coli* strains in a semi-solid agar.

The bacteriological motility test in semi-liquid agar did not reveal differences between the motile strains M17 and ATCC43890 (Fig. 1). Both strains spread in semi-liquid agar with similar effectiveness, which was noticeably higher than that of the control non-motile strain JM109. The opacity areas showing bacterial spreading in the semi-liquid agar after 24 h of growth were 4 ± 0.5 mm for JM109, which could be regarded as a result of cell division (Fig. 1). The difference in opacity areas between M17 and ATCC43890 of 51 ± 4 mm and 50 ± 2 mm, respectively, was insignificant (*p* > 0.05). Thus, the obtained results showed that the probiotic and virulent strains had the same macroscopic motility.

### E. coli strains had different motility patterns

**Figure 2:**
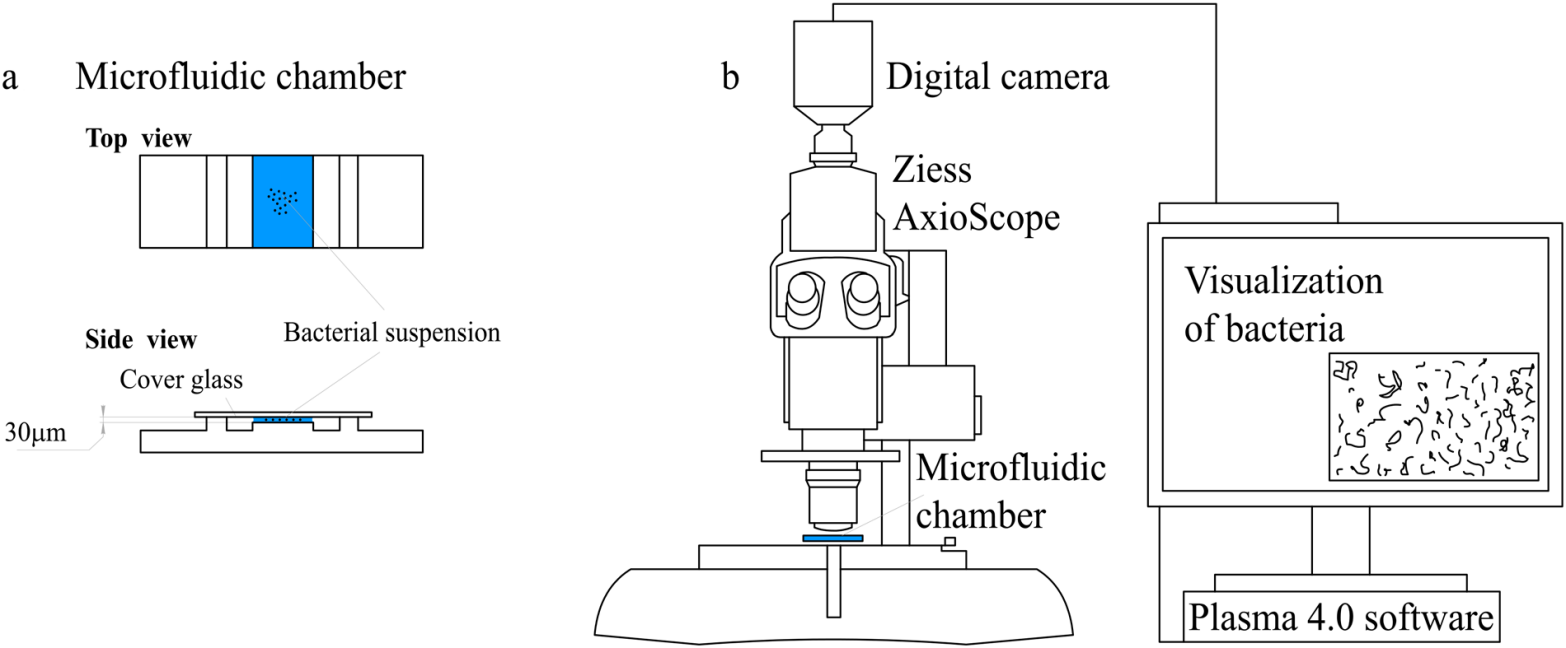
Scheme of (**a**) microfluidic chamber and (**b**) experimental setup.

A microfluidic chamber was designed to study bacterial movement. The chamber had an area of 8 × 10 mm and a height of 30 µm, covered with a glass slide (Fig. 2). The centre of the chamber was filled with a bacterial suspension with an OD_600_ of approximately 2.0.

**Figure 3:**
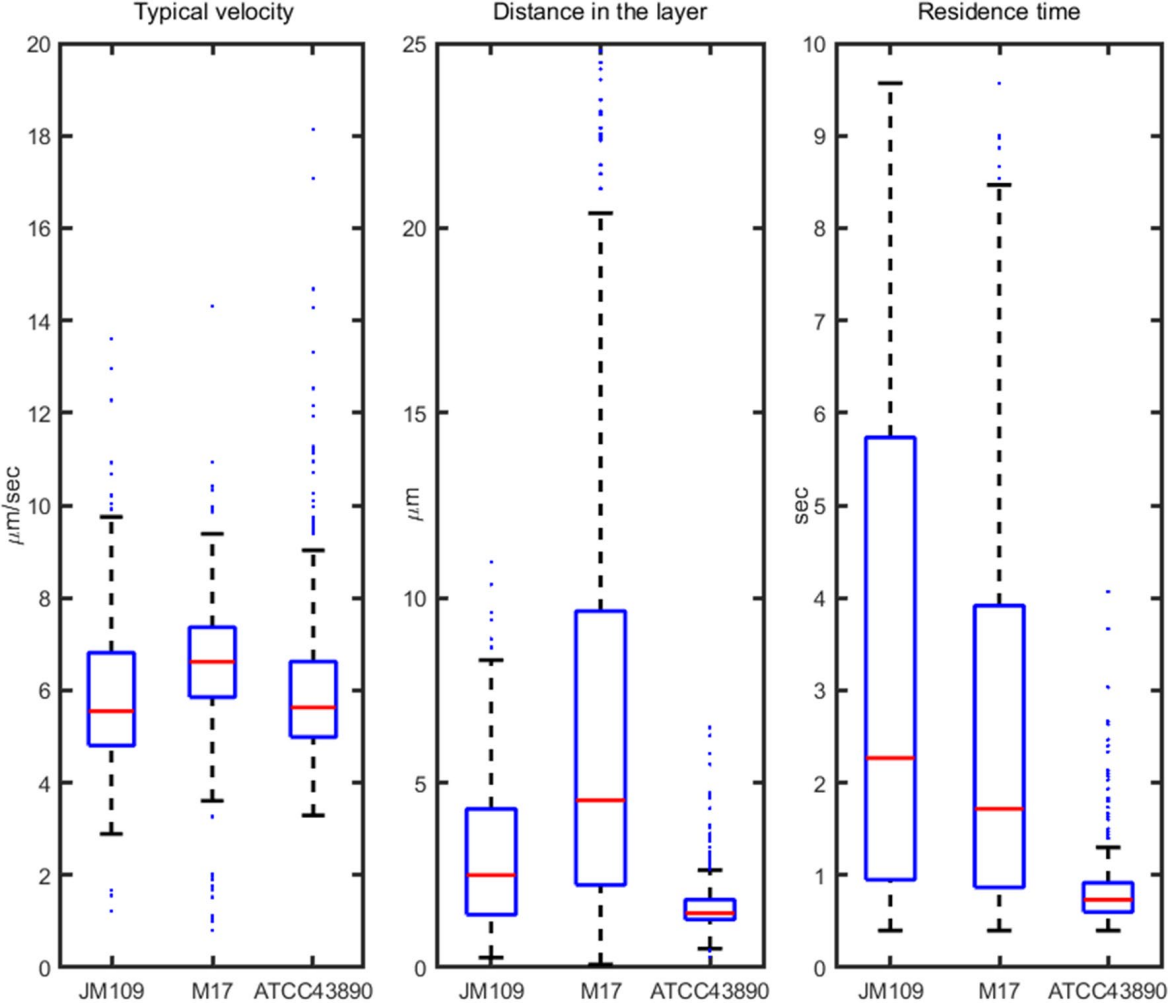
Boxplot with standard whiskers (1.5 IQR) of main characteristics of *E. coli* strains’ movement: typical bacterium velocity (left), typical distance covered by bacteria within the layer (middle), typical residence time spent in the layer (right). Red lines—median values for characteristics. All distributions consist of ~ 200 trajectories.

**Figure 4:**
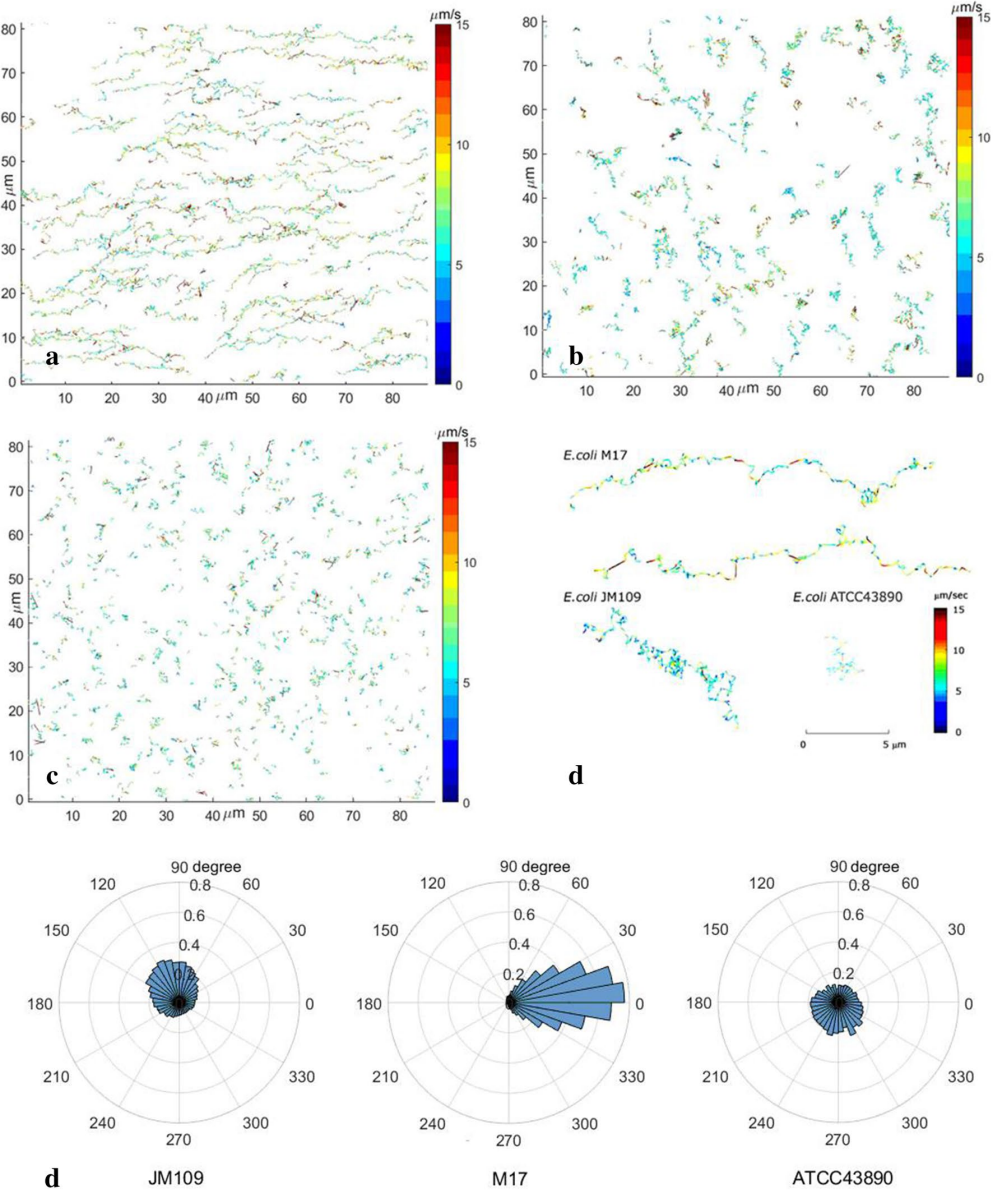
Reconstructed trajectories, color-coded according to the values of instantaneous velocities, for different strains of *E. coli*: (**a**) M17 (**b**) JM109 (**c**) ATCC43890. (**d**) Typical trajectory for each strain. (**e**) Angle distributions of instantaneous velocity vectors for each strain. M17 exhibits pronounced collective motion in one direction.

We observed bacterial movement in the middle layer of the bacterial suspension, that is, at a distance of 10–15 μm from both the chamber bottom and the glass (Fig. 2a). This distance was used to ensure that the observed motion characterised the landing stage and was not due to bacterial interactions with the surface. To capture movement patterns, we recorded a set of short videos that were processed to obtain the main characteristics of movement (Fig. 3) and reconstruct trajectories within the layer of observation (Fig. 4). Based on the obtained data, we revealed differences in the movement patterns between strains.

Average values of velocity, distance and residence time (i.e., the time spent by an individual bacterium within the observed liquid layer) were calculated based on the results of ~ 200 reconstructed trajectories in 10 s video, taken for each strain (see Materials and Methods). Based on macroscopic observations, we suggested that the non-motile JM109 would demonstrate Brownian movement, while the movement of the two other strains would characterise the motion of motile bacteria showing parameters different from the non-motile strain.

Boxplot of typical bacterium velocity (Fig. 3 left) showed only a little difference between strains with mean velocity values of 5.93 μm/sec (CI 95% 5.7–6.16), 6.49 μm/s (CI 95% 6.26–6.72), and 6.36 μm/s (CI 95% 5.93–6.79) for JM109, M17, and ATCC43890, respectively (Anova, F = 4.56, *p* < 0.05). At this, difference of mean values was significant only between JM109 and M17 (Tukey, HSD = 0.58, *p* < 0.05). While the speed values were almost similar for all three strains, other characteristics were strikingly different (Fig. 3, middle, right). The average distances, which individual bacterium ran within horizontal layer before leaving, were 3.17 μm (CI 95% 2.88–3.46), 7.01 μm (CI 95% 6.17–7.86), and 1.83 μm (CI 95% 1.65–2) for JM109, M17, and ATCC43890, respectively (Anova, F = 81.54, *p* < 0.01). Interestingly, motile ATCC43890 showed almost a twice shorter mean distance than the poorly motile JM109. An average residence time was 3.62 s (CI 95% 3.19–4.05), 2.99 s (CI 95% 2.62–3.36), and 0.94 s (CI 95% 0.84–1.04) for JM109, M17, and ATCC43890, respectively (Anova, F = 51.5, *p* < 0.01). The time spent within the observed layer by the M17 and JM109 strains was approximately the same, suggesting that both strains left the layer due to stochastic processes and gravity. Strain ATCC439890 demonstrated the shortest residence time compared to JM109 and M17 (Tukey, HSD = 2.68 (and 2.05), *p* < 0.01). These data may be explained if we suggest that ATCC439890 preferred to ‘scan’ layer by layer, moving in a vertical direction more frequently than other strains. More statistical details one can see in a Supplementary.

Figure 4 shows ~ 200 bacteria trajectories obtained in one experiment that are color-coded according to the values of instantaneous velocities. The poorly motile JM109 strain demonstrated a typical Brownian-like motion, i.e., its chaotic movements were due to stochastic collisions with ambient molecules of liquid (Fig. 4b,d). The absence of a preferred movement direction is typical for Brownian particles as well^23^. M17 strain, on the contrary, had longer, less chaotic tracks with pronounced directional movement (Fig. 4a,d). At the same time, the ATCC43890 trajectories were the shortest, as if they had just crossed through the observed layer, and did not have a pronounced collective or individual directional movement, similar to JM109 (Fig. 4c,d). To check these observations, polar histograms, which show the direction of the instantaneous velocity vectors averaged over all bacteria, were constructed (Fig. 4e). The polar histograms showed that the population of M17 bacteria had a preferred instantaneous movement direction while other strains did not. These observations suggested that a noticeable part of the M17 bacteria moved in the same direction within each particular moment.

### *E. coli* suspension in terms of active matter: M17 demonstrated superdiffusive behavior while ATCC43890 demonstrated subdiffusive behavior

To better understand the movement characteristics of each strain, we analysed bacterial behaviour in terms of the active matter theory^24^. From this point of view, bacterial suspension can be considered an active matter, that is, a system consisting of active particles capable of converting chemical energy to directional motion.

**Figure 5:**
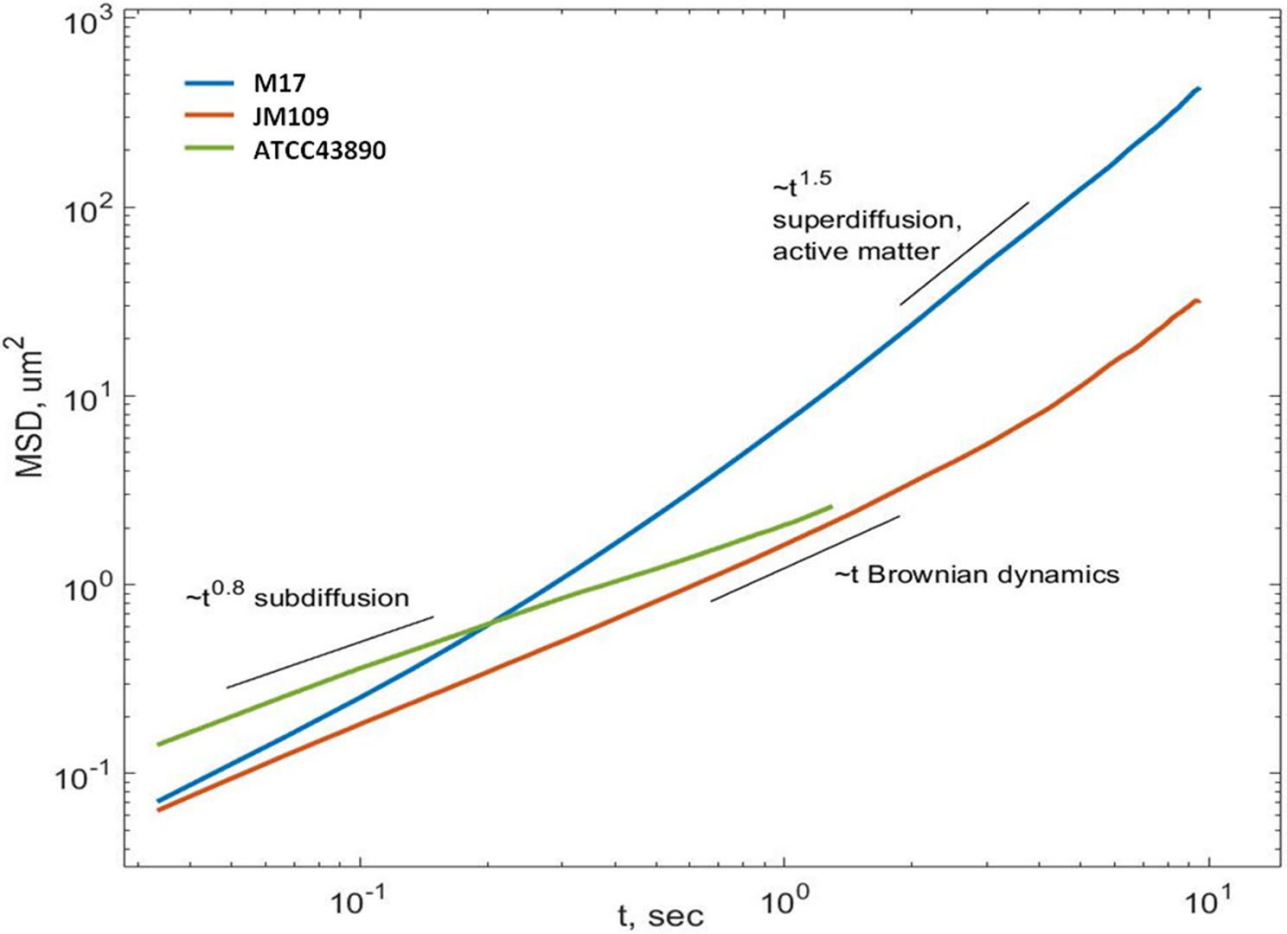
Log–log time dependence of mean square displacement (MSD) for various strains of *E*. *coli*.

The analysis in terms of active matter theory is based on the calculation of the time (*t*) dependence of the mean square displacement (MSD). This method reveals prevailing patterns of motion, varying from conventional Brownian (chaotic) motion up to the ballistic regime. Figure 5 shows a graph of MSD(*t*) on a log–log scale for the *E. coli* strains. Calculation of the MSD time dependence for the poorly motile strain JM109 revealed a linear MSD time dependence (Fig. 5). The linear MSD(*t*) dependence is characteristic of the motion of passive particles colliding with molecules of the medium, which is typical for classical Brownian dynamics^23^. For strain M17, the mean square displacement was proportional to the time taken to a power of 1.5 (~ t^1.5^). Such dependence indicates the superdiffusion of bacteria in the suspension^24^. Superdiffusion means that directional movement in the horizontal plane predominates over the chaotic component of motion, and M17 bacteria can be classified as active Brownian particles. These data are in line with the direct calculation of the average velocity direction vector (Fig. 4e).

The asymptotics for ATCC43890 turned out to be equal to MSD ~ t^0.8^ as if the bacteria had subdiffusive behaviour. However, we had to consider that the experimental design was restricted to observing bacteria in a horizontal layer ~ 5 μm thick. At the same time, the total height of the microfluidic camera was 30 μm, so the bacteria could quickly leave the field of visibility deep into the solution. In other words, our experimental design allowed observation of the horizontal motion only, while bacteria moved in the 3D medium, and subdiffusive bacterial motion in the horizontal direction might be compensated by superdiffusion in the vertical direction. The suggestion about the preferential movement of ATCC43890 bacteria in the vertical direction is supported by a short distance and short residence time in the horizontal layer (Fig. 4).

Therefore, the active matter analysis supported the individual characteristics of the motion of the studied *E. coli* strains. JM109 showed mostly Brownian movement, M17 moved collectively, representing active Brownian particle behaviour, while ATCC43890 moved highly likely in a vertical direction. Considering these noticeable differences in landing motility patterns between the studied strains, we were interested in how these microscopic characteristics would influence macroscopic parameters such as adhesion.

### Motile and non-motile strains demonstrated different dynamics of adhesion to the plastic surface

**Figure 6:**
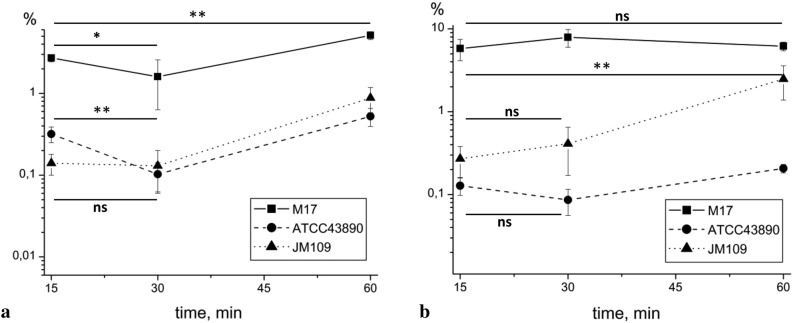
Efficiency of adhesion among different *E. coli* strains to (**a**) plastic and (**b**) HEp-2 cell line. ns—not significant, *p < 0.05, **p < 0.01. Additional information related to statistical analysis see in a Supplementary.

Next, we compared the effectiveness of bacterial adhesion to horizontal surfaces in the three studied strains. Bacteria were added to a 24-well plate, and the number of adhered cells was determined after 15, 30, and 60 min of incubation. In 15 min, poorly motile JM109 showed the lowest adhesion rate (0.14 ± 0.04% of initially added bacteria), but the prolongation of exposition to 60 min significantly increased the efficiency of its adhesion by a factor of 6.3 ± 2.1 times (Tukey, HSD = 2.49, *p* < 0.01) (Fig. 6a). Motile strains demonstrated more effective fast adhesion with adhered 2.71 ± 0.2% and 0.32 ± 0.07% for M17 and ATCC43890, respectively, 15 min post bacterial addition. However, the dynamics of motile strain adhesion were completely different from those of JM109. After 60 min of incubation, the absolute number of attached M17 bacteria doubled and reached 5.12 ± 0.56% of the initial amount (Tukey, HSD = 2.31, *p* < 0.01). For ATCC43890, increasing the incubation time to 30 min resulted in a three-fold decrease in the number of attached cells from 0.32% to 0.1% (Tukey, HSD = 0.22, *p* < 0.01), and 60 min incubation caused a 0.52% adhesion efficiency, which was the lowest among the three strains and 1.7 times lower than that of the poorly motile strain JM109. Thus, adhesion seemed to be a dynamic process for the motile strains and the result of a passive settlement for the poorly motile strain.

### Differences in motility patterns resulted in different efficiency of adhesion to human HEp-2 cells

Both M17 and ATCC43890 strains can colonise the intestine as the first strain is probiotic, and the second is pathogenic. To analyse whether the observed differences in motility patterns influence bacterial interactions with epithelial cells, we tested bacterial adhesion to human adenocarcinoma HEp-2 cells, which are often used as models for *E. coli* infection^25,26^. They have laminin and fibronectin receptors, which are specific targets for the hemorrhagic *E. coli* strain ATCC43890^27^. HEp-2 cells were grown up to a monolayer, and bacteria were added at a multiplicity of infection MOI of 1:100. Adhesion efficiency was tested 15, 30, and 60 min post infection by direct plating of adhered bacteria as described in the Materials and Methods.

After 15 min of incubation, adhesion rates were similar to the data obtained for the plastic surface for JM109 and M17, but not for ATCC43890 (0.27 ± 0.11% and 5.79 ± 1.56% for JM109 and M17, respectively) (Fig. 6b). ATCC43890 adhesion to HEp-2 cells was lower than to the plastic surface (0.13 ± 0.03% and 0.32 ± 0.07% for HEp-2 and plastic surfaces 15 min post bacterial addition, respectively). Incubation for 60 min caused a 9.2 fold increase in the number of adhered JM109 bacteria compared to the first 15 min (Tukey, HSD = 2.31, *p* < 0.01). In contrast, prolonged incubation for 30—60 min did not increase the number of adhered M17 and ATCC43890 bacteria (1.07 and 1.5 fold increase, respectively, compared to bacteria adhered after 15 min incubation, *p* > 0.05).

The results suggested that the increase in the adhesion efficiency of the poorly motile *E. coli* strain JM109 after 60 min of incubation was mainly due to bacterial sedimentation under the influence of gravity. The absence of positive dynamics for motile strains might suggest that the percentage of bacteria able to interact with human cells was limited, so such bacteria adhered to the cells during the first 15 min, and later the population was exhausted. Alternatively, the number of available receptors on the cell surface may be restricted. Motile bacteria seized all receptors within 15 min, and later, there were no free receptors for bacteria to adhere. The difference in adhesion dynamics to the plastic surface and HEp-2 cells supported the second suggestion: while the number of M17 and ATCC43890 bacteria adhered to the plastic surface increased from 15 to 60 min, it was almost constant when bacteria interacted with HEp-2 cells.

### Differences in motility patterns resulted in different patterns of adhesion

The obtained results demonstrated that the M17 and ATCC43890 strains demonstrated a noticeable difference in adhesion efficiency 15 min after bacterial addition. This difference was observed for adhesion to both plastic surfaces and human cells; therefore, this difference was not due to the varying abundance of strain-specific surface receptors used by bacteria to adhere to cells. We suggest that different motility patterns may affect the adhesion efficiency of motile strains.

**Figure 7:**
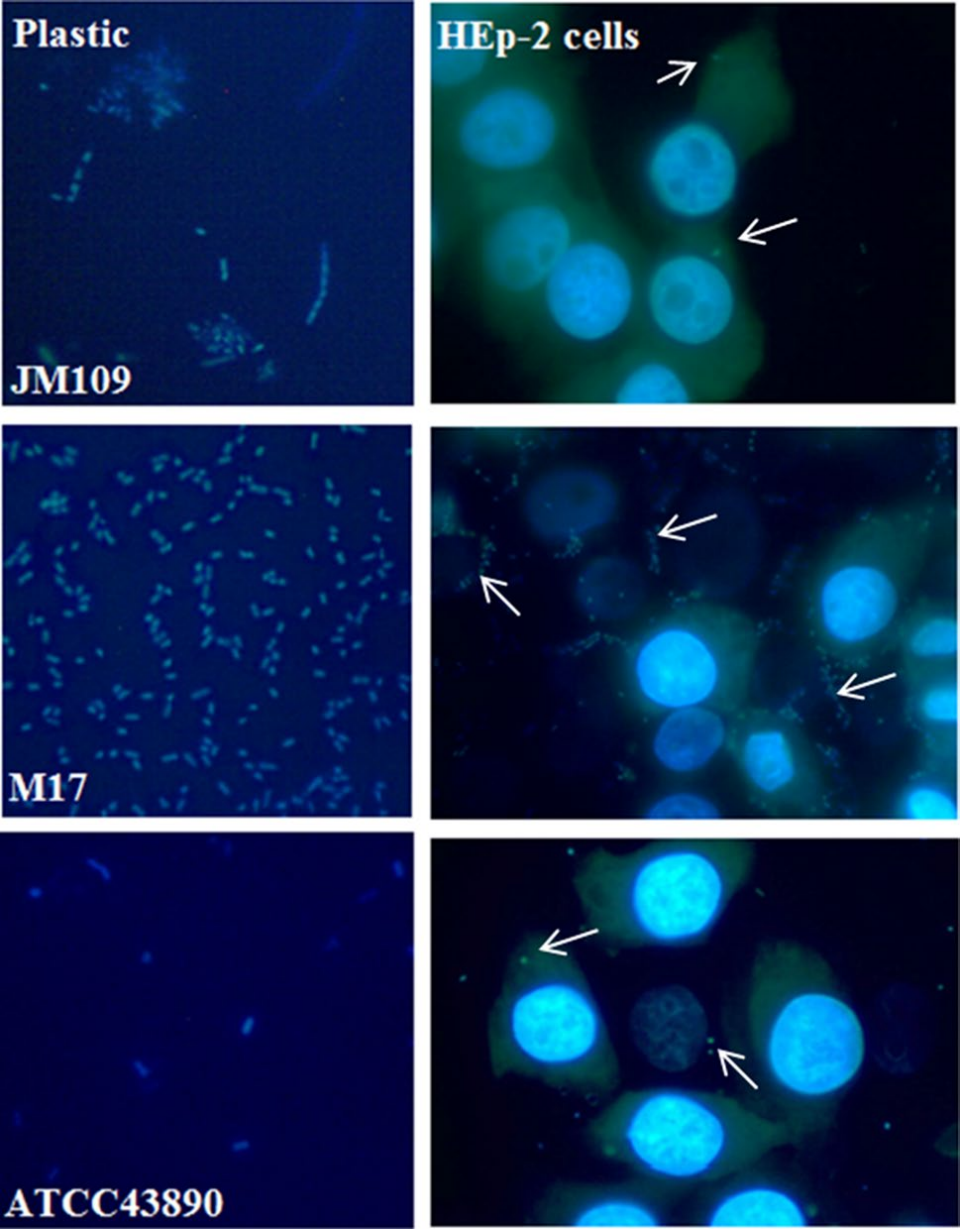
The microphotographs of adhered *E. coli* strains. Arrows show the adhered bacteria on specific locations of cell surfaces.

To better understand the events during adhesion, we made microphotographs of bacteria adhered to plastic and HEp-2 cell surfaces after 15 min of incubation (Fig. 7). Poorly motile JM109 adhered to plastic and HEp-2 surfaces as small auto-aggregates that suggested initial flocculation, which is in line with our suggestion about passive sedimentation of JM109 cells (Fig. 7a,d). M17 demonstrated a non-uniform distribution on the plastic surface with linear clusters of 3–5 or more bacterial cells (Fig. 7b). Interacting with HEp-2 cells, M17 bacteria concentrated predominantly at the border of neighbouring cells in groups of more than ten bacteria with the same linear geometry as at the plastic surface (Fig. 7e). In contrast, ATCC43890 bacteria had completely different surface distributions. Single or groups of two bacteria were observed on the plastic surface, and mainly isolated bacteria were observed on the HEp-2 cell surface (Fig. 7c). ATCC43890 bacteria were distributed uniformly over surfaces, and there were no observed tropisms toward cell–cell contact areas for ATCC43890 bacteria, in contrast to M17 bacteria. The amount of *E. coli* ATCC43890 that adhered to the cells was higher than that of JM109, which contradicted the results of the plating experiment (see Fig. 6b). This discrepancy could be due to the weak adhesion strength of the ATCC43890 strain and the more delicate washing of the samples for taking microphotographs.

Taken together, the obtained results demonstrated noticeable differences between the motile strains not only in adhesion efficiency but also in surface distribution.

## Discussion

In this study, three *E. coli* strains were compared from the point of view of specific features characterising bacteria leaving the bulk and approaching the solid surface. This initial stage of bacterial interactions with the surface, known as the landing stage, is important for further choice between adhesion to the surface or continuing the movement^28^. Three strains were compared: two motile strains, the probiotic strain M-17 and virulent O157:H7 strain ATCC43890, and the poorly motile laboratory strain JM109. Bacterial motion patterns were studied at a distance of 10–15 μm from the surface, which is a distance characteristic for the landing stage^13^.

The parameters of the average and maximal distances and residence time demonstrated by strain JM109 were considered characteristic of Brownian movement because JM109 is known to be poorly motile due to a deletion of *the rec*A gene^14–17^. Low JM109 motility was confirmed by a macroscopic motility test. We expected that motile bacteria would move parallel to the surface, demonstrating longer average and maximal distances and a shorter residence time than JM109. This suggestion was based on the well-known models of bacterial landing and near-surface motility^29,30^. These models suggest that *E. coli* swimming at a distance of 20 μm or less from the surface reorients the cells for their axis to be parallel to the surface due to hydrodynamic interactions^29,30^. Indeed, the probiotic strain M17 demonstrated this behaviour. M17 bacteria moved along lengthy, horizontally oriented trajectories.

Surprisingly, the probiotic strain M17 was the only strain that moved predictably in our experiments, while the motile virulent strain ATCC43890 did not demonstrate long trajectories. Moreover, ATCC43890 showed average and maximal distances and residence times shorter than the poorly motile strain JM109. These data suggest that ATCC43890 quickly left the horizontal liquid layer and moved preferentially in the vertical direction. Previously, Ipina et al. also used the enterohemorragic *E.coli* strain serotype O157:H7 to investigate its movement patterns near a glass surface^12^. They observed smooth circular trajectories due to the interruption of bacterial movement by short stop events (‘transient adhesion events’)^12^. It was concluded that these trajectories provided a faster exploration of a surface that could be profitable for all bacteria. We did not observe such behaviour because we focused on the landing stage, excluding the interaction of bacteria with a surface. Still, further observations on ATCC43890 movement with the techniques that allowed observation of the vertical motion are required to support the suggestion about preferable vertical motion direction.

The next parameter we analysed was the traits of the collective behaviour of bacteria. From a physical point of view, motile bacteria are active Brownian particles that can convert external energy into kinetic energy of motion^26–31^. Based on this definition, the suspension of motile bacteria is a special case of active media that can be characterised by the formation of zones with aligned dynamic clusters, whirls, and jets, especially with increasing bacterial concentration^32,33^, similar to^33,34^ what we observed aligning the velocity vectors of M17 (Fig. 3). We did not find any published data on the subdiffusive behaviour of a motile bacterium, which was demonstrated by the strain ATCC43890; in addition, we failed to find data on a collective motion in a vertical direction that we suggested for this strain. This phenomenon requires further study.

We considered that the observed motility difference between probiotic and virulent strains could correlate with the efficiency of their adhesion to plastic and eukaryotic cells. Taken together, plating and microscopic data suggested that poorly motile JM109 tended to self-aggregate, form non-shaped flocs by a few cells, and floc sedimentation due to gravity. This result seems trivial. M17 adhesion in the form of linear formations was a more interesting observation. These linear formations were observed on both the plastic surfaces and the cell monolayer. When M17 bacteria interacted with a HEp-2 cell monolayer, the linear formation was strictly associated with the cell-to-cell border. Therefore, our first suggestion was that M17 bacteria specifically interacted with receptors located at the intercellular junctions. However, this suggestion was contradicted to the observation of similar structures formed on the plastic surfaces. To explain the linear patterns of M17 adhesion, we suggested that the linear character of adhesion was a direct consequence of the collective horizontal unidirectional movement demonstrated by this strain. We assumed that this collective unidirectional movement caused the formation of local ‘braking zones’ when interactions with a certain obstacle stopped the movement of at least one bacterium. Any obstacle that slows down or stops the movement of one bacterium should lead to the formation of a row of slow-moving or blocked bacteria, that is, an ‘adhesion zone’ (Fig. 7a,b). Such collective adhesion must increase the effectiveness of surface colonisation. Similar behaviour was observed for *Salmonella* Typhimurium^7^. Similar adhesion patterns were previously described for saprophytic *E. coli* strains by Frommel et al., who referred to it as a global adhesion strategy for commensal *E. coli*^35^.

The obstacles that stop bacterial rows can be quite minor irregularities on a plastic surface. When intercellular contact sites in the epithelial cell monolayer were considered, scanning electron microscopy revealed vertical edges formed by intercellular junctions^36^. Although these edges were approximately 200 nm, this may be enough to slow down the speed of at least one bacterium because of the vertical character of the edge that prevents bacteria from moving around the obstacle. As a result, explosive growth in the number of bacteria in this zone can be observed. Therefore, we speculate that the horizontally oriented collective movement determines the characteristics of adhesion and the high adhesion efficiency.

The ATCC43890 bacteria demonstrated a different character of the movement and uniform distribution of single or double cells on plastic and cell surfaces. We suggest that this type of adhesion could be a consequence of the vertical character of the movement. Considering that virulent ATCC43890 requires a ligand-receptor interaction, this behaviour could be beneficial for the search of receptors. This ‘vertical’ movement may also be promising for intracellular pathogens. It has been shown, for example, that *S*. Typhimurium selects spherical mitotic cells for invasion^7^, and there is a correlation between the number of neighbouring eukaryotic cells and the efficiency of bacterial invasion^37^. The angle of interaction between bacteria and the surface is also important. For intestinal pathogens, the penetration of the mucus layer covering the surface of cells is critical, and motility facilitates this process^38^. It is evident that the shortest trajectory, in this case, is down through the protective layer at an angle close to 90°. Recently, Otte et al. showed that the active movement of *S.* Typhimurium, but not chemotaxis, was the primary strategy for the search of host cells because the adhesion was a random process depending on bacterial motility parameters^39^.

**Figure 8:**
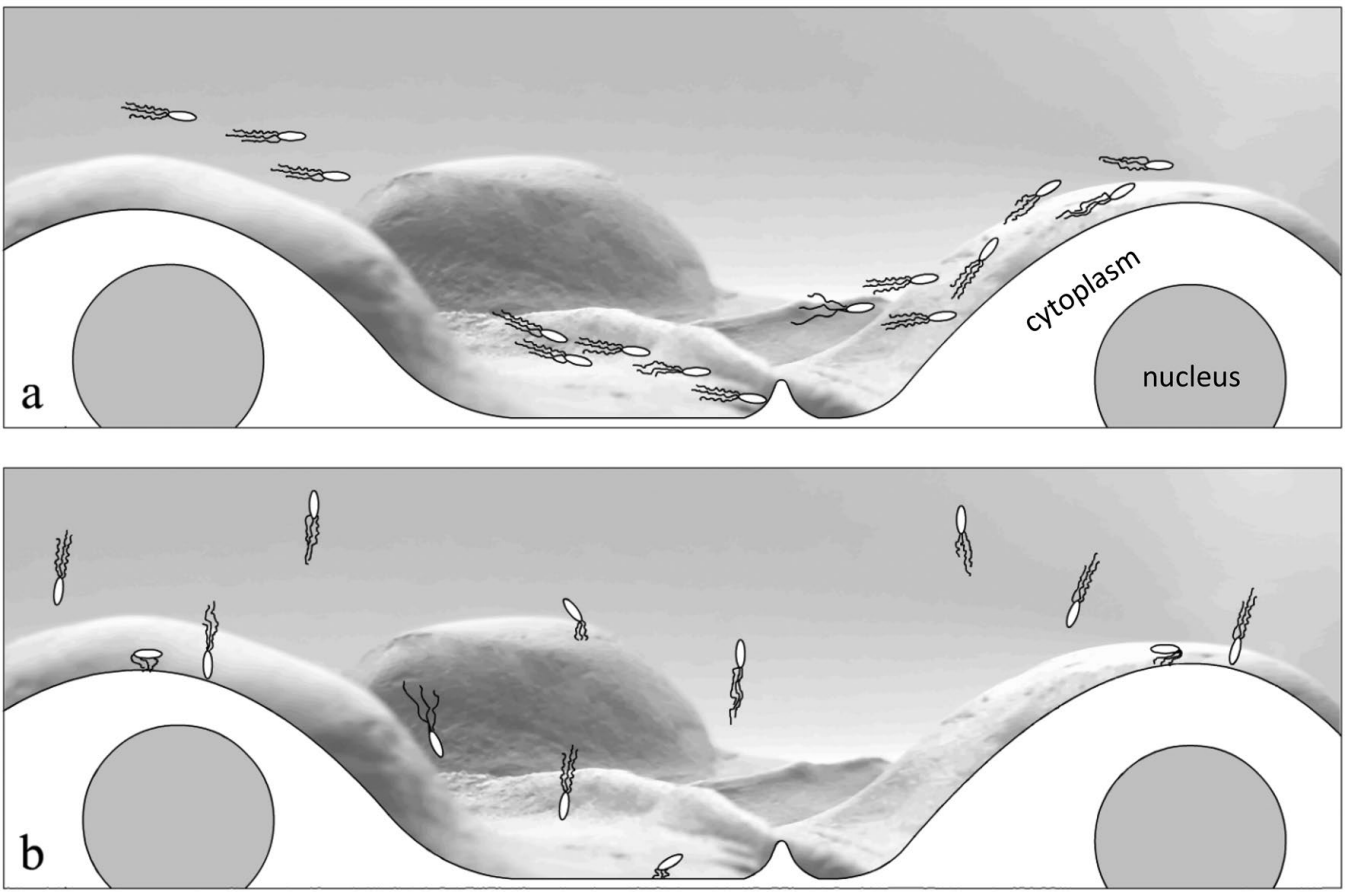
Horizontal (**a**) and vertical (**b**) movement for surface adhesion. Horizontal strategy implies collective directional motion mainly in the horizontal layer with gradual slow sedimentation and a non-uniform ‘row’ pattern of adhesion close to surface irregularities. Vertical strategy implies non-directional motion in the mainly vertical direction and uniformly distributed adhesion patterns.

Thus, based on the data obtained, we propose two bacterial motility patterns for adequate adhesion: horizontal and vertical movements at the landing stage. The horizontal movement, characterised by a collective directed movement of microorganisms within the horizontal layer, could allow more effective non-specific surface colonisation. Movement in a vertical direction may promote a faster transition from bulk to near-surface swimming, allowing microorganisms to ‘test’ the surface and increase the probability of interaction with the receptor (Fig. 8). Therefore, individual differences in motility patterns may underlie different adhesion strategies.

## Materials and methods

### Bacterial strains and growth conditions

The investigation of bacterial movement was conducted with three *E. coli* strains from the Gamaleya National Research Centre collection: the enterohemorrhagic *E. coli* ATCC43890 strain ATCC43890, the probiotic strain *E. coli* M17, and *E. coli* JM109 with poor motility. Bacteria were routinely grown on Luria–Bertani (LB) agar (Amresco, USA) media at 37 °C.

### Detection of bacterial motility on semi-solid agar

Semi-solid LB medium (0.5% agar) was dispensed in Petri dishes. Three *E. coli* strains were inoculated into semi-solid agar using a stab method with a straight needle. After 24 h incubation at 37 °C, a photograph of the Petri dish was taken, and the diameters of the opaque area were measured. This experiment was conducted in triplicates.

### Microfluidic chamber construction

The prototype of the microfluidic chamber was a counting chamber that was modified by reducing the height of the medium layer to 30 μm (Fig. 1). The chamber was created by 3D printing of a transparent resin (Anycubic Photon, China).

### Observation of bacterial motility

For the investigation of bacterial movement, the liquid overnight culture that was grown in a shaking incubator at 37 °C was centrifuged, and the sediment was resuspended in sterile phosphate-buffered saline (PBS) to reach an optical density of 2 (OD_600_ = 2), which corresponded to a concentration of 1 × 10^9^ cells/mL.

The bacterial suspension was inserted into the microfluidic chamber, followed by video visualisation at 1000-fold magnification. The chamber was placed onto the slide table of the Zeiss Axio Scope A1 microscope equipped with a digital camera DCM510 (China), which transmitted video images with a resolution of 1280 × 960 px (Fig. 2). The sample preparation and visual observation took approximately 10–15 min, and then a series of 30 s videos were made at a speed of 30 frames/sec. The size of the observed field of view was ~ 81 × 86 mm^2^, and the depth of field was 5–10 microns.

At least five videos for each sample were recorded, and all experiments were conducted five times.

### Video image processing and analysis

For the processing and analysis of videos, approximately 10 s of the video stream was cut out. We used the videos without obvious signs of movement of the bacteria due to a laminar flow to be sure that the bacteria move freely. he selected videos were processed using specially developed software Plasma 4.0, in which a statistical subtraction of the background, Fourier filtering of the image, and frame-by-frame identification of the location of each of the bacteria were performed. We considered trajectories longer than ten frames to cut off the ‘false bacteria’ blur circles with short trajectories of 1–10 frames in length that did not disappear during the preprocessing.

Thus, we obtained the main characteristics of the movement of bacteria: (1) positions of the bacteria in each frame, (2) instantaneous and average values of the speeds, (3) trajectory and mean square displacement, and (4) characteristic time and characteristic length of run of bacteria in the observing layer.

### Adhesion of *E. coli* strains to HEp-2 cells

HEp-2 cells were cultivated in Dulbecco's Modified Eagle Medium (DMEM; Paneco, Russia) supplemented with 10% fetal bovine serum (GIBCO) and no antibiotics. Cells were cultured at 37 °C and 5% CO_2_.

The cells were seeded in a 24-well plate. While achieving an average number, 300 000 cells/well, the nutritious medium was replaced with 1 mL *E. coli* suspension at a ratio of 1:100. After 15, 30, and 60 min of incubation, the bacterial suspension was removed, and the surface of the wells was rinsed thrice with sterile PBS. To destroy the eukaryotic cells, PBS was removed, and 100 μl of 1% Triton X-100 was added to the wells. After 15 s, when the cell membranes disappeared, 900 μl PBS was added, and the suspension was resuspended thoroughly. After that, the samples were diluted and quantitated by plating on appropriate agar plates.

All the experiments were made in triplicate thrice.

### Adhesion of *E. coli* strains to plastic

The adhesion efficiency was tested on a polyethylene terephthalate (PET) surface of 24-well plates. The same concentrations and volumes of bacterial suspension, as we used to investigate adhesion to HEp-2 cells, were added to each well of a 24-well plate. After 15, 30, and 60 min of incubation, the bacterial suspension was removed, and the surface of the wells was rinsed thoroughly thrice with sterile PBS. Bacteria were then removed from the surface with a sterile cotton swab. Tenfold dilutions were plated, and the number of colony-forming units (CFU) was counted after 18 h of incubation at 37 °C.

All the experiments were conducted in triplicate thrice.

### Bacterial staining

Bacteria adhered to the plastic and HEp-2 cells were washed three times with sterile PBS, fixed with 3.7% neutral formaldehyde, and stained with Hoechst 33,342 (ThermoFisher, USA) according to the manufacturer's protocol. Fluorescence microscopy and 1000 × magnification were used to visualise the samples.

### Statistical analysis

Values are expressed as mean ± SD. Statistical analysis was performed using a one-way ANOVA with post hoc Tukey’s test. Statistical differences were considered significant when the *p*-value was < 0.05.

## Supplementary Information

Supplementary Information.
