# Supplementary material for: Strain specific motility patterns and surface adhesion of virulent and probiotic Escherichia coli
Source: Sci Rep. 2022 Jan 12;12:614. doi: 10.1038/s41598-021-04592-y (PMC8755817; doi:10.1038/s41598-021-04592-y)
Supplement: Supplementary file 1 — Supplementary Information. [file 41598_2021_4592_MOESM1_ESM.pdf]

Supplementary information of
"Strain specific motility patterns and surface adhesion of virulent and probiotic *Escherichia coli* "

Abdulkadieva M.M., Sysolyatina E.V., Vasilieva E.V., Gusarov A.I., Domnin P.A., Slonova D.A., Stanishevskiy Y.M., Vasiliev M.M., Petrov O.F., Ermolaeva S.A.

We used one-way ANOVA method (F-statistics) with post hoc Tukey's test (HSD - statistics).

Summary of statistical analysis:

			**Anova**		**Tukey HSD test** (pairwise comparisons)		
		parameter	F	p-value	**JM109:M17**	**JM109:ATCC43890**	**M17:ATCC43890**
Motion characteristics		<V>	4.56	p<0.05	p < 0.05	insignificant	insignificant
		<L>	81.54	p<0.01	p < 0.01	p < 0.01	p < 0.01
		<t>	51.49	p<0.01	p < 0.05	p < 0.01	p < 0.01
					**15min:30min**	**15min:60min**	**30min:60min**
Adhesion efficiency, %	to plastic	**JM109**	68.37	p<0.01	insignificant	p < 0.01	p < 0.01
		**M17**	41.44	p<0.01	p < 0.05	p < 0.01	p < 0.01
		**ATCC43890**	30.78	p<0.01	p < 0.01	p < 0.01	p < 0.01
	to HEp-2 cell	**JM109**	56.05	p<0.01	insignificant	p < 0.01	p < 0.01
		**M17**	3.52	p<0.05	insignificant	insignificant	insignificant
		**ATCC43890**	19.92	p<0.01	insignificant	p<0.01	p<0.01

Extended version of obtained results:

I) Main characteristics of bacteria *E. coli* strains' motion in the layer (see Figure 3)

a) **Average velocity**: statistical difference is significant (Anova, F = 4.56, $p<0.05$) only for strains JM109 and M17.

**Summary of Data**				
	Measures			
	**JM109**	**M17**	**ATCC43890**	Total
N	222	240	166	628
∑X	1316.8156	1563.3561	1055.2576	3935.4293
Mean	5.9316	6.514	6.357	6.267
∑X2	8495.3151	11003.2887	8009.9529	27508.5567
Std.Dev.	1.7599	1.8518	2.8088	2.1308

**Result Details**				
Source	SS	df	MS	
Between-treatments	40.9571	2	20.4786	**F = 4.56164**
Within-treatments	2805.8097	625	4.4893	**p=0.0108**
Total	2846.7668	627		

**Post Hoc Tukey HSD**				
Pairwise Comparisons		HSD.05 = 0.4927<br>HSD.01 = 0.6132	Q.05 = 3.3224<br>Q.01 = 4.1354	
**JM109:M17**	M1 = 5.93<br>M2 = 6.51	0.58	Q = 3.93<br>(p = .01557)	p<0.05
**JM109:ATCC43890**	M1 = 5.93<br>M3 = 6.36	0.43	Q = 2.87<br>(p = .10626)	**insignificant**
**M17:ATCC43890**	M2 = 6.51<br>M3 = 6.36	0.16	Q = 1.06<br>(p = .73457)	**insignificant**

b) **Average distance in the layer**: all three strains have statistically significant difference (Anova, F=81.54, $p<0.01$).

**Summary of Data**				
	Measures			
	**JM109**	**M17**	**ATCC43890**	Total
N	222	240	166	628
∑X	703.5422	1683.4739	303.2013	2690.2174
Mean	3.1691	7.0145	1.8265	4.284
∑X2	3314.6288	22256.9262	777.4695	26349.0245
Std.Dev.	2.2158	6.6118	1.1643	4.8625

Result Details				
Source	SS	df	MS	
Between-treatments	3067.7734	2	1533.8867	**F = 81.54158**
Within-treatments	11756.9361	625	18.8111	**p=1E-16**
Total	14824.7095	627		

Post Hoc Tukey HSD				
Pairwise Comparisons		HSD.05 = 1.0085<br>HSD.01 = 1.2553	Q.05 = 3.3224<br>Q.01 = 4.1354	
**JM109:M17**	M1 = 3.17<br>M2 = 7.01	3.85	Q = 12.67<br>(p = .00000)	p<0.01
**JM109:ATCC43890**	M1 = 3.17<br>M3 = 1.83	1.34	Q = 4.42<br>(p = .00524)	p<0.01
**M17:ATCC43890**	M2 = 7.01<br>M3 = 1.83	5.19	Q = 17.09<br>(p = .00000)	p<0.01

c) **Average residence time in the layer**: all three strains have statistically significant difference (Anova, F=51.49, p<0.01).

Summary of Data				
	Measures			
	**JM109**	**M17**	**ATCC43890**	Total
N	222	240	166	628
∑X	803.1667	717.7	156.1666	1677.0333
Mean	3.6179	2.9904	0.9408	2.67
∑X2	5234.7635	4123.1679	213.4944	9571.4258
Std.Dev.	3.2463	2.8761	0.6352	2.8501

Result Details				
Source	SS	df	MS	
Between-treatments	720.48	2	360.24	**F = 51.49184**
Within-treatments	4372.5371	625	6.9961	**p=1E-16**
Total	5093.017	627		

Post Hoc Tukey HSD				
Pairwise Comparisons		HSD.05 = 0.6150<br>HSD.01 = 0.7655	Q.05 = 3.3224<br>Q.01 = 4.1354	
**JM109:M17**	M1 = 3.62<br>M2 = 2.99	0.63	Q = 3.39<br>(p = .04432)	p<0.05
**JM109:ATCC43890**	M1 = 3.62<br>M3 = 0.94	2.68	Q = 14.46<br>(p = .00000)	p<0.01
**M17:ATCC43890**	M2 = 2.99<br>M3 = 0.94	2.05	Q = 11.07<br>(p = .00000)	p<0.01

II) Efficiency of adhesion of bacteria *E. coli* strains to plastic (see Figure 6a)

a) for JM109:

**Summary of Data**				
	Measures			
	**15 min**	**30 min**	**60 min**	Total
N	6	8	5	19
∑X	0.8486	1.0852	12.8617	14.7955
Mean	0.1414	0.1357	2.5723	0.779
∑X2	0.1293	0.2674	35.5094	35.9061
Std.Dev.	0.0431	0.131	0.7786	1.1639

**Result Details**				
Source	SS	df	MS	
Between-treatments	21.8305	2	10.9153	**F = 68.37695**
Within-treatments	2.5541	16	0.1596	**p=1E-07**
Total	24.3847	18		

**Post Hoc Tukey HSD**				
Pairwise Comparisons		HSD.05 = 0.5902<br>HSD.01 = 0.7740	Q.05 = 3.6491<br>Q.01 = 4.7855	
**15min : 30min**	M1 = 0.14<br>M2 = 0.14	0.01	Q = 0.04<br>(p = .99966)	**insignificant**
**15min : 60min**	M1 = 0.14<br>M3 = 2.57	2.43	Q = 15.03<br>(p = .00000)	p<0.01
**30min : 60min**	M2 = 0.14<br>M3 = 2.57	2.44	Q = 15.06<br>(p = .00000)	p<0.01

b) for M17:

**Summary of Data**				
	Measures			
	**15 min**	**30 min**	**60 min**	Total
N	8	6	5	19
∑X	21.7274	9.6723	25.1237	56.5234
Mean	2.7159	1.612	5.0247	2.975
∑X2	59.5979	20.3374	127.2172	207.1524
Std.Dev.	0.2897	0.9742	0.4944	1.472

**Result Details**				
Source	SS	df	MS	
Between-treatments	32.6897	2	16.3449	**F = 41.44264**
Within-treatments	6.3104	16	0.3944	**p=1E-05**
Total	39.0001	18		

Post Hoc Tukey HSD				
Pairwise Comparisons		HSD.05 = 0.9277<br>HSD.01 = 1.2167	Q.05 = 3.6491<br>Q.01 = 4.7855	
**15min : 30min**	M1 = 2.72<br>M2 = 1.61	1.1	Q = 4.34<br>(p = .01896)	p<0.05
**15min : 60min**	M1 = 2.72<br>M3 = 5.02	2.31	Q = 9.08<br>(p = .00002)	p<0.01
**30min : 60min**	M2 = 1.61<br>M3 = 5.02	3.41	Q = 13.42<br>(p = .00000)	p<0.01

c) for ATCC43890:

Summary of Data				
	Measures			
	**15 min**	**30 min**	**60 min**	Total
N	6	6	8	20
∑X	1.9112	0.6155	4.1857	6.7124
Mean	0.3185	0.1026	0.5232	0.336
∑X2	0.635	0.0743	2.3208	3.0301
Std.Dev.	0.0723	0.0473	0.1367	0.2023

Result Details				
Source	SS	df	MS	
Between-treatments	0.6091	2	0.3045	**F = 30.78068**
Within-treatments	0.1682	17	0.0099	**p=1E-06**
Total	0.7773	19		

Post Hoc Tukey HSD				
Pairwise Comparisons		HSD.05 = 0.1411<br>HSD.01 = 0.1844	Q.05 = 3.6280<br>Q.01 = 4.7418	
**15min : 30min**	M1 = 0.32<br>M2 = 0.10	0.22	Q = 5.55<br>(p = .00296)	p<0.01
**15min : 60min**	M1 = 0.32<br>M3 = 0.52	0.2	Q = 5.26<br>(p = .00457)	p<0.01
**30min : 60min**	M2 = 0.10<br>M3 = 0.52	0.42	Q = 10.82<br>(p = .00000)	p<0.01

III) Efficiency of adhesion of bacteria *E. coli* strains to HEp-2 cell line (see Figure 6b)

a) for JM109:

**Summary of Data**				
	Measures			
	**15 min**	**30 min**	**60 min**	Total
N	15	13	6	34
∑X	4.1195	5.3815	14.8887	24.3896
Mean	0.2746	0.414	2.4814	0.717
∑X2	1.3386	2.9744	42.2991	46.6121
Std.Dev.	0.1217	0.2495	1.0348	0.9393

**Result Details**				
Source	SS	df	MS	
Between-treatments	22.8087	2	11.4043	**F = 56.04792**
Within-treatments	6.3077	31	0.2035	**p=1E-05**
Total	29.1164	33		

**Post Hoc Tukey HSD**				
Pairwise Comparisons		HSD.05 = 0.5049<br>HSD.01 = 0.6446	Q.05 = 3.4806<br>Q.01 = 4.4434	
**15min : 30min**	M1 = 0.27<br>M2 = 0.41	0.14	Q = 0.96<br>(p = .77706)	**insignificant**
**15min : 60min**	M1 = 0.27<br>M3 = 2.48	2.21	Q = 15.21<br>(p = .00000)	p<0.01
**30min : 60min**	M2 = 0.41<br>M3 = 2.48	2.07	Q = 14.25<br>(p = .00000)	p<0.01

b) for M17:

**Summary of Data**				
	Measures			
	**15 min**	**30 min**	**60 min**	Total
N	13	9	5	27
∑X	75.3681	71.1723	30.8763	177.4168
Mean	5.7975	7.908	6.1753	6.571
∑X2	470.2365	611.1877	192.8916	1274.316
Std.Dev.	1.6655	2.4585	0.7453	2.0429

**Result Details**				
Source	SS	df	MS	
Between-treatments	24.6489	2	12.3244	**F = 3.52702**
Within-treatments	83.8629	24	3.4943	**p=0.454**
Total	108.5118	26		

**Post Hoc Tukey HSD**				
Pairwise Comparisons		HSD.05 = 2.3743<br>HSD.01 = 3.0559	Q.05 = 3.5317<br>Q.01 = 4.5456	
**15min : 30min**	M1 = 5.80<br>M2 = 7.91	2.11	Q = 3.14<br>(p = .08806)	**insignificant**
**15min : 60min**	M1 = 5.80<br>M3 = 6.18	0.38	Q = 0.56<br>(p = .91694)	**insignificant**
**30min : 60min**	M2 = 7.91<br>M3 = 6.18	1.73	Q = 2.58<br>(p = .18376)	**insignificant**

c) for ATCC43890:

**Summary of Data**				
	Measures			
	**15 min**	**30 min**	**60 min**	Total
N	10	9	8	27
∑X	1.2802	0.7743	1.653	3.7076
Mean	0.128	0.086	0.2066	0.137
∑X2	0.1792	0.0844	0.3464	0.61
Std.Dev.	0.0412	0.0471	0.0263	0.0623

**Result Details**				
Source	SS	df	MS	
Between-treatments	0.063	2	0.0315	**F = 19.92411**
Within-treatments	0.0379	24	0.0016	**p=1E-06**
Total	0.1009	26		

**Post Hoc Tukey HSD**				
Pairwise Comparisons		HSD.05 = 0.0470<br>HSD.01 = 0.0605	Q.05 = 3.5317<br>Q.01 = 4.5456	
**15min : 30min**	M1 = 0.13<br>M2 = 0.09	0.04	Q = 3.16<br>(p = .08596)	**insignificant**
**15min : 60min**	M1 = 0.13<br>M3 = 0.21	0.08	Q = 5.91<br>(p = .00095)	p<0.01
**30min : 60min**	M2 = 0.09<br>M3 = 0.21	0.12	Q = 9.06<br>(p = .00000)	p<0.01
